# Supplementary material for: QuickEd: high-performance exact sequence alignment based on bound-and-align
Source: Bioinformatics. 2025 Mar 13;41(3):btaf112. doi: 10.1093/bioinformatics/btaf112 (PMC11937955; doi:10.1093/bioinformatics/btaf112)
Supplement: btaf112_Supplementary_Data [file btaf112_supplementary_data.pdf]

Supplementary material for QuickEd: High-performance exact sequence alignment based on bound-and-align

Max Doblas, Oscar Lostes-Cazorla, Quim Aguado-Puig,
Cristian Iñiguez, Miquel Moreto, and Santiago Marco-Sola

S1 Experimental Evaluation

S1.1 Tools and Environment

We evaluated the performance of QuickEd against the following state-of-the-art sequence alignment libraries/algorithms libraries.

- **Scrooge:** Commit `f478f54` at `https://github.com/CMU-SAFARI/Scrooge`
- **Edlib:** Commit `931be2b` at `https://github.com/Martinsos/edlib`
- **KSW2:** Commit `289609b` at `https://github.com/lh3/ksw2`
- **A* Pairwise Aligner:** Library featuring *A*PA* and *A*PA2*. Using commit `8ca0f21` at `https://github.com/RagnarGrootKoerkamp/astar-pairwise-aligner`
- **WFA2-lib:** Library featuring both *WFA* and *BiWFA*. Commit `42f8bf3` at `https://github.com/smarco/WFA2-lib`

Edlib is integrated into the `align_benchmark`(`https://github.com/maxdoblas/QuickEd/tree/benchmark/tools`, commit `8a0c58e`) tool, as a ground-truth comparison. A* Pairwise Aligners is also integrated through its C binding. WFA/BiWFA can be executed through QuickEd's integration or using the WFA2-lib repository. Scrooge and KSW2 can be found already integrated into the WFA2-lib repository (`benchmark` branch, commit `455be3b` (`https://github.com/smarco/WFA2-lib/tree/455be3bf8ba43f4fd7a33017b306a1bb5d8dcbec`)), and they have been evaluated using its `align_benchmark` tool which is very similar to ours.

All the experiments were run on a machine with the following specifications.

- **CPU:** Intel Xeon Platinum 8480+ @ 3GHZ *(fixed frequency)*
- **RAM:** 256 GB

All runs were performed using a single thread and a single, exclusive node (enforced by SLURM using `#SBATCH --hint=nomultithread` and `#SBATCH --exclusive`).

We use the following versions for compilers and other tools:

- **GCC:** 12.3.0
- **CMake:** 3.25.1
- **Rust:** 1.85.0-nightly

With this setup, AVX512 instructions are available whenever the tool can leverage them. All libraries and tools were compiled following the instructions provided in their respective repositories.

Regarding other tools and libraries, we excluded from the evaluation other libraries such as Parasail (Daily, 2016) and SeqAn (Döring *et al.*, 2008) as they were not designed to align very long and noisy sequences and failed to complete the executions. Also, we excluded SneakySnake (Alser *et al.*, 2020) from the evaluations because it acts as a sequence filter and does not produce the alignment or the score.

Regarding other drops (e.g., X-drop and Z-drop) and adaptive banded strategies, note that these heuristics are not guaranteed to find the optimal alignment. Therefore, a direct comparison with QuickEd is not entirely appropriate, as these methods aim for different outcomes. However, these techniques can complement QuickEd and have potential applications within QuickEd's bounding step to improve performance. For instance, X-drop could be leveraged to terminate some bounding steps early, reducing its overhead. Similarly, adaptive banded alignment could replace the current BandEd algorithm used in QuickEd-Bound, potentially enhancing accuracy while maintaining the same execution time.

S1.2 Datasets

To evaluate the QuickEd library, we used real datasets produced by Illumina, PacBio, and Oxford Nanopore technologies. Table S1 shows each dataset's number of sequences, minimum, average, and maximum sequence length and error rate. Illumina 250 dataset was obtained from NIST's Genome in a Bottle (GIAB) project and can be found at https://github.com/genome-in-a-bottle/giab_data_indexes. PacBio HiFi dataset was obtained from PrecisionFDA Truth Challenge V2 and can be found at https://precision.fda.gov/challenges/10. ONT UltraLong dataset, aligned against the v1.1 CHM13 assembly, was obtained from Bowden *et al.* (2019). ONT PromthION dataset was obtained from the Human Pangenome Reference Consortium (Miga and Wang (2021)). ONT MiniION-a dataset contains sequences produced by Oxford Nanopore MiniION and was obtained from https://github.com/pairwise-alignment/pa-bench/releases/download/datasets/ont-500k.zip. All sequences are longer than 500kbps. MiniION-b dataset contains sequences produced by Oxford Nanopore MiniION from Bowden *et al.* (2019) aligned against the v1.1 CHM13 assembly. All sequences are longer than 500kbps and contain genetic variation and large gaps. More in detail, Figure S1 shows sequence length and error distribution for each real dataset.

Moreover, simulated datasets have also been used for development and experimentation purposes. These datasets were generated using our generate_dataset tool, which can be found at https://github.com/maxdoblas/QuickEd/tree/main/tools/generate_dataset. This tool generates synthetic random datasets with fixed length and uniformly distributed error. Table S2 shows the used sequence lengths and the number of sequences for each length. For all lengths, we produced datasets with error rates of 1%, 5%, 10% and 20%. All simulated datasets have been uploaded to Zenodo and can be found at https://doi.org/10.5281/zenodo.14526793.

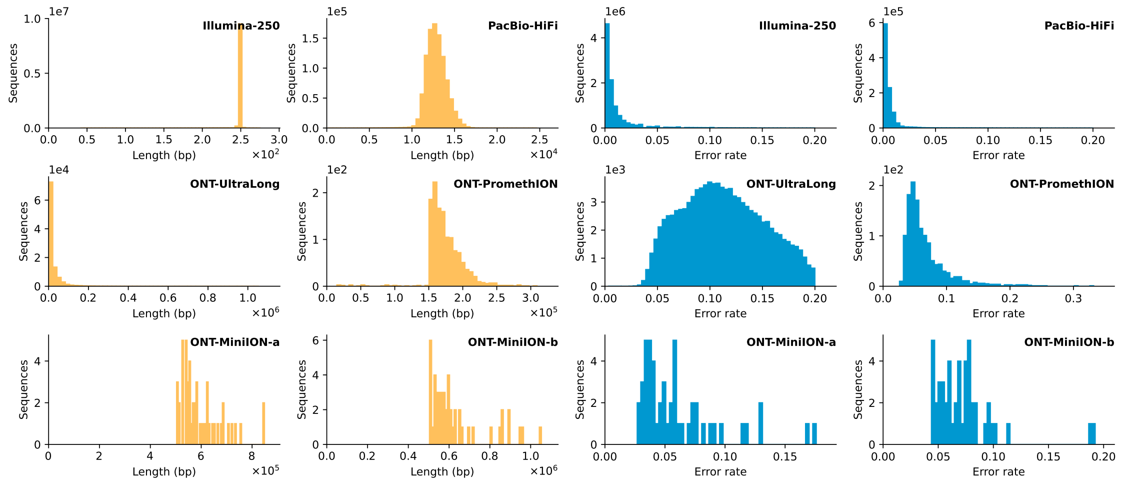

Figure S1: Sequence length (yellow) and error distribution (blue) of all real datasets used in the experimental evaluation.

Table S1: Properties of real datasets used in the experimental evaluation.

Dataset	No. pairs	Seq. Length (bps)			Error rate (%)		
		min	avg	max	min	avg	max
Illumina 250	100M	140	248	275	0.0	1.0	20.0
PacBio HiFi	10M	201	12,847	24,640	0.0	0.6	20.0
ONT UltraLong	100k	125	21331	1,053,101	0.3	11.3	20
ONT PromethION	1312	14,749	174,219	309,277	2.6	6.7	33.2
ONT MiniION-a	50	502,992	597,582	848,888	2.7	6.1	17.6
ONT MiniION-b	48	503,480	635,931	1,053,101	4.4	7.4	19.2

S2 QuickEd Time Analysis

QuickEd has two main building blocks: a score-bounding algorithm (bound step) and a score-bounded alignment algorithm (align step). Given a sequence pair $p \in P$, from a set of input sequence pairs P, the score-bounding algorithm approximates the alignment-score ($\hat{s}_p$). This estimation may be sub-optimal but serves as a guide to narrow down the alignment search space during the score-bounded align step, reducing the overall execution time.

$$T = \sum_{p \in P} T_B(p) + \sum_{p \in P} T_A(p, \hat{s}_p) \quad (1)$$

The overall execution time (T) is the aggregate of the score-bounding time (T_B) and the score-bounded alignment time (T_A), using $\hat{s}_p$ bound, as shown in Equation 1. Generally, more precise score-bounding algorithms tend to be more time-consuming. However, the execution time of score-bounded alignment algorithms depends on the alignment-score estimation provided by the bounding step. In particular, a score-bounded alignment algorithm becomes more costly if the estimation provided by the bound step is less accurate. Thus, an important trade-off exists between the precision attained during the bound step and the overall execution time (i.e., the sum of the bound and align execution time).

Table S2: Characteristics of simulated datasets used in the experimental evaluation.

Seq. Length (bps)	10k	100k	500k	1M
No. pairs	10k	1k	200	200

S2.1 Quicked's Cascade of Score-Bounding Components

Designing a single score-bounding algorithm that simultaneously delivers good speed and accuracy across all types of sequences is challenging. A more practical approach involves implementing a cascade of multiple score-bounding algorithms (called components), each tailored to handle sequences of different characteristics. This score-bounding is structured hierarchically, progressing from faster, less accurate components to slower, more precise ones. Figure S2 shows a n-step score-bounding algorithm with n different components $(B_1, B_2, ..., B_n)$ connected in cascade. The initial component (B_1) should be a fast and simple score-bounding algorithm able to quickly handle highly similar sequences, leaving $B_{2..n}$ components to process dissimilar sequences. Sequence pairs are processed sequentially through various components until one component produces a sufficiently accurate estimation or, in the worst case, a final score-bounding component B_n is reached. This B_n component executes a more time-consuming algorithm but produces an exact alignment score. As a result, this cascading approach balances computational efficiency and accuracy, attending to the diverse characteristics of input sequences.

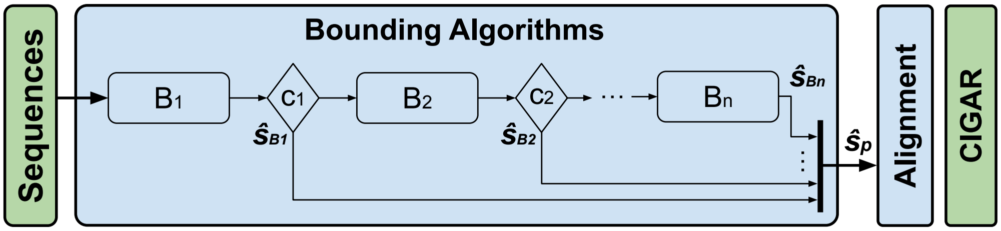

Figure S2: Block diagram of n-components cascade of score-bounding algorithms.

S3 WindowEd Parameter Exploration

We have performed a design space exploration of the window and overlap size parameters of the WindowEd algorithm to select the configuration of the WindowEd(S) and WindowEd(L) components used in QuickEd. We have evaluated the execution time and recall of the different configurations of W window size and O overlap. It is worth noting that QuickEd implements a vectorized implementation of the smallest WindowEd configuration (i.e., $W = 128$ and $O = 64$). Figure S3 shows the accuracy and execution time of each WindowEd configuration when aligning real datasets.

The analysis shows that the smaller WindowEd configuration ($W = 128$, $O = 64$) delivers the highest performance, being $1.7 - 2.1\times$ faster than the next smallest configuration ($W = 192$, $O = 64$), while only sacrificing 9% of recall in the worst-case. Consequently, we choose to utilize the $W = 128$ and $O = 64$ configuration as the WindowEd(S) component to efficiently score-bound highly-similar sequences. For the WindowEd(L) component, we choose a window size of $W = 640$ with an overlap $O = 128$. In practice, this configuration shows a good balance between execution time and accuracy, minimizing the execution time of the WindowEd(L) while reducing the number of sequences processed by the exponential BandEd (i.e., last component).

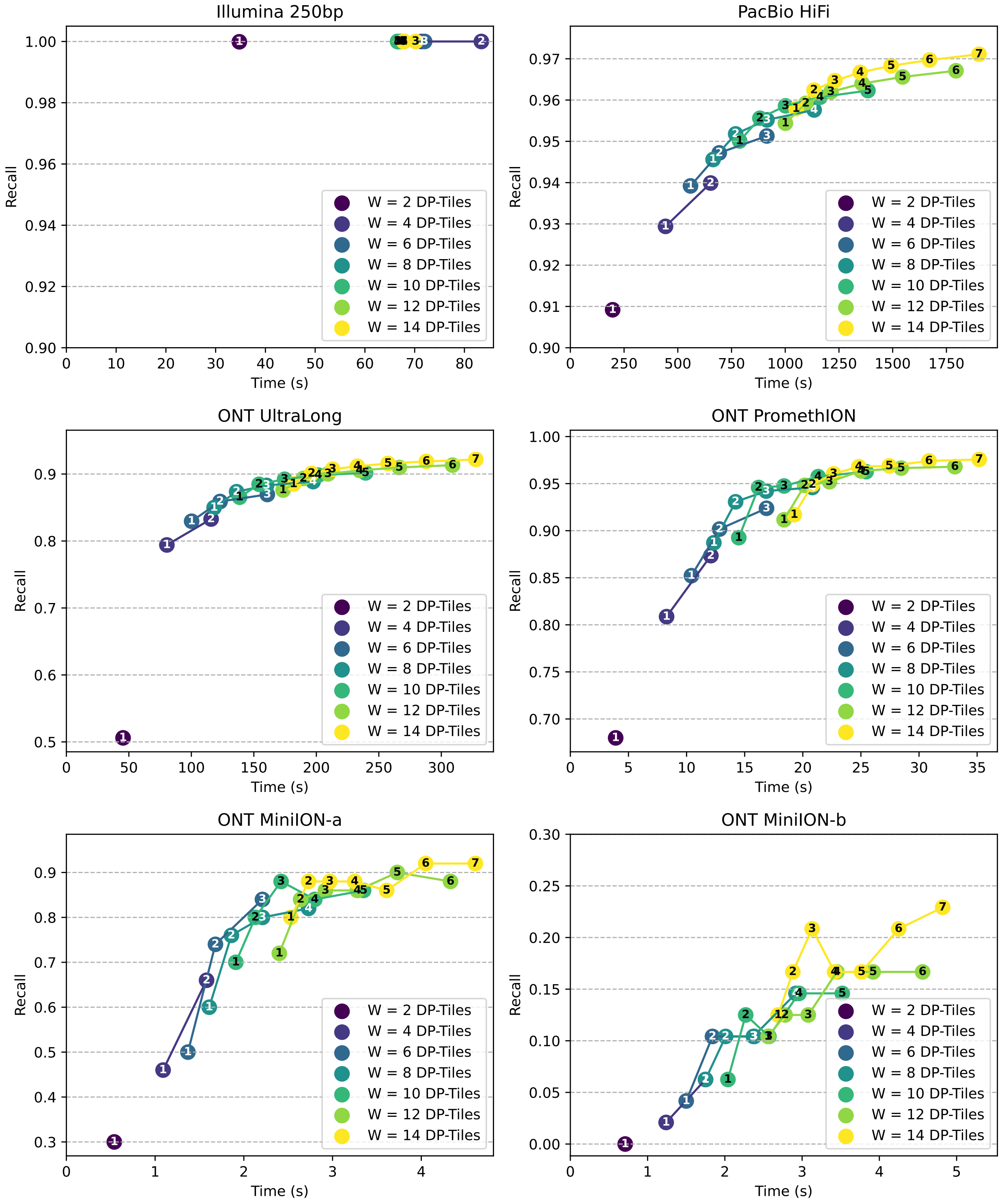

Figure S3: Design space exploration of the W window size and O overlap size parameters of the WindowEd algorithm. The W and O sizes are expressed in blocks of 64x64 DP-elements. The number inside the markers (ⓧ) represents the overlap size in blocks.

S4 Experimental Evaluation of Exact Algorithms using Simulated and Real Datasets

Table S3: Time (in seconds) and memory usage (in megabytes) for the different exact alignment tools aligning simulated and real datasets. Executions over 48 hours are labelled as "t.o." (time out) and executions that run out of memory as "o.o.m.".

		Time (s)						Memory (MB)					
		KSW2	WFA	BiWFA	A*	Edlib	QuickEd	KSW2	WFA	BiWFA	A*	Edlib	QuickEd
10 Kbp	1%	1036.2	0.3	0.4	25.4	29.2	2.6	199.4	4.2	4.4	6.8	4.0	6.4
	5%	10607.4	1.7	2.1	29.4	56.7	6.8	19547.1	32.2	10.1	26.6	5.2	18.8
	10%	o.o.m	8.7	10.7	38.9	147.6	21.5	o.o.m	134.0	27.0	100.8	8.3	24.4
	20%	o.o.m	19.9	22.5	44.3	250.8	37.5	o.o.m	442.4	34.1	186.9	13.1	32.3
100 Kbp	1%	1035.6	3.6	4.4	24.0	34.3	3.7	199.3	6.0	5.2	6.8	4.0	7.4
	5%	10620.0	33.8	42.5	27.5	141.0	19.7	19561.5	132.6	8.5	24.7	5.1	17.2
	10%	o.o.m	180.0	207.0	36.1	460.8	79.2	o.o.m	2242.8	18.0	96.1	8.4	23.6
	20%	o.o.m	372.0	417.6	42.1	879.0	150.6	o.o.m	8975.3	38.7	185.7	13.3	32.4
500 Kbp	1%	1036.8	12.3	15.8	32.7	40.7	5.2	199.7	28.7	5.2	6.6	4.0	8.6
	5%	10588.2	124.8	152.4	39.3	238.2	32.9	19566.7	362.6	7.3	24.7	5.0	24.8
	10%	o.o.m	627.0	758.4	50.9	869.4	145.8	o.o.m	7925.1	23.5	88.1	8.6	18.4
	20%	o.o.m	1270.2	1576.8	59.2	1696.2	282.0	o.o.m	32117.6	37.1	172.2	13.3	32.3
1 Mbp	1%	1034.4	38.4	52.0	1666.2	51.8	8.0	199.5	73.9	5.1	74.1	4.0	12.5
	5%	10624.2	401.4	516.6	31389.0	431.4	58.0	19577.8	1040.6	7.5	3388.3	5.2	16.8
	10%	o.o.m	1996.8	2585.4	t.o.	1675.2	262.8	o.o.m	25587.5	24.2	t.o.	8.5	22.0
	20%	o.o.m	4100.4	5332.8	o.o.m	3309.0	515.4	o.o.m	106322.6	38.9	o.o.m	13.5	32.8
Illumina 250bp		594.0	4.3	9.1	636.6	186.0	60.6	4.5	3.9	3.8	4.4	3.9	3.8
PacBio HiFi		t.o.	58.8	79.2	10341.6	3405.6	400.8	t.o.	90.4	5.8	255.2	5.3	24.5
ONT UL		o.o.m	2824.8	3313.8	o.o.m	3858.6	835.2	o.o.m	116534.2	29.5	o.o.m	13.8	33.7
ONT PromethION		42892.8	353.4	411.0	19974.0	574.2	96.6	140280.9	10440.4	9.8	53528.2	6.6	27.9
ONT UUL		o.o.m	157.2	172.2	6915.0	214.2	39.1	o.o.m	48303.0	19.2	213988.7	11.9	29.0
ONT MiniION		o.o.m	226.8	258.0	o.o.m	286.8	67.8	o.o.m	116634.2	24.0	o.o.m	15.6	33.6

S5 QuickEd Execution Time Distribution along the Different Components

QuickEd's current implementation uses an alignment-score bounding algorithm composed of 3 components connected in cascade: WindowEd(S), WindowEd(L), and BandEd (i.e., an exponential band implementation). QuickEd's alignment-score bounding algorithm stops when a component's estimation is accurate enough. Hence, not all the components in the cascade are executed for every input sequence pair.

Table S4 shows the number of sequences processed by each component within QuickEd's alignment-score bounding algorithm. For the Illumina, PacBio, and ONT PromethION datasets, the WindowEd(S) component can process more than 95% of the sequences. By incorporating the WindowEd(L) component, we generate accurate estimations for 99-100% of the sequences. Consequently, utilizing the exponential BandEd component is only necessary to estimate approximately 0.6% of the PacBio sequences, notably reducing the performance overhead of this accurate but expensive component. However, the ONT UltraLong and MiniION datasets, characterized by having noisier sequences with longer gaps, require more accurate and time-consuming components (i.e., WindowEd(L) and BandEd)

Table S4: Number of sequences processed by each component. Note that all the sequences have to be processed by the WindowEd(S) component (first component in the bound step) and the QuickEd-Align algorithm (align step).

	QuickEd-Bound			QuickEd-Align
	WindowEd(S)	WindowEd(L)	BandEd	
Illumina 250bp	10M (100%)	22.1k (0.22%)	0 (0%)	10M (100%)
PacBio HiFi	1M (100%)	38.0k (3.8%)	5.9k (0.6%)	1M (100%)
ONT UltraLong	100k (100%)	41k (41%)	2k (2%)	100k (100%)
ONT PromethION	1312 (100%)	59 (4.5%)	0 (0%)	1312 (100%)
ONT MiniION-a	50 (100%)	13 (26%)	2 (4%)	50 (100%)
ONT MiniION-b	48 (100%)	45 (93.8%)	14 (29.1%)	48 (100%)

Figure S4 shows the time distribution of the different components inside QuickEd for the different datasets. As expected, QuickEd-Align execution time dominates. However, in the datasets with shorter sequences (Illumina and PacBio), the execution time of the WindowEd(S) bounding component is still significant. Aligning datasets containing short sequences, the number of DP-elements computed by the WindowEd(S) component is similar to the ones computed by QuickEd-Align since the band tends to be very small. In contrast, the execution time of WindowEd(S) using ONT datasets is negligible since the bandwidth used within QuickEd-Align is much larger.

The execution time of the most accurate components (i.e., BandEd and WindowEd(L)) impacts the overall execution time aligning PacBio, ONT UltraLong, and ONT MiniION datasets. This is due to the necessity of using these components to estimate alignment-scores for many sequences in these datasets. Conversely, in the Illumina and ONT PromethION datasets, where the number of sequences processed by each component is either zero or nearly zero, the impact on execution time is minimal or negligible. These results are expected after analyzing the number of sequences processed by the different components Table S4.

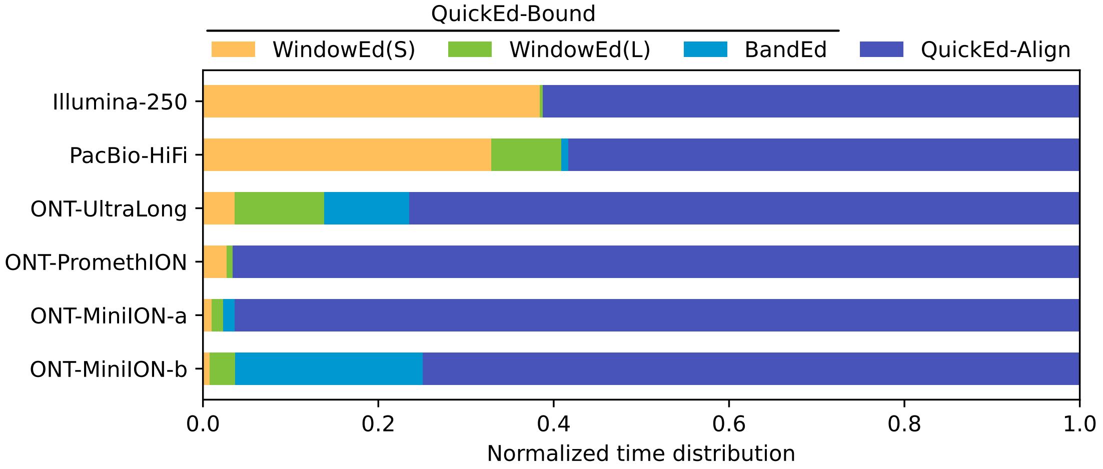

Figure S4: Normalized time distribution of the different components inside QuickEd for the different datasets.

S6 Vectorization in QuickEd

The core components of QuickEd are vectorized to improve performance by leveraging the SIMD units that are now standard in most modern CPUs. After analyzing the execution time breakdown of QuickEd (Fig S4), we observed that the components consuming the majority of execution time in QuickEd are the BandEd score-only and WindowEd(S) operations. The BandEd score-only operation is employed in both QuickEd-Bound and QuickEd-Align to bound the error and to identify breakpoints during Hirschberg's algorithm, respectively. Meanwhile, the WindowEd(S) operation exhibits a significant execution time when processing sequences with low error rates.

S6.1 BandEd Score Only Vectorization

The vectorization of the BandEd alignment follows a commonly used strategy where elements from each antidiagonal are calculated within the same vector. In this case, since we use the BPM approach, multiple 64-bit words are computed in parallel, each representing vectors of DP-elements. In this case, the computation pattern of the DP-matrix remains unchanged computing blocks of 64×64 DP-elements (i.e., 64 words of 64 DP-elements) are computed column by column. However, a SIMD vector processes 4 blocks in parallel (i.e., AVX2), significantly improving performance.2

Moreover, the Banded score-only operation does not require storing all values of the DP-matrix. Instead, we only store the right border of each block to facilitate the computation of the next column and the bottom element of the SIMD vector for the computation of the next block within the same column. This approach eliminates the need for gather and scatter memory operations, which are usually costly in terms of latency and bandwidth, allowing a higher performance.

S6.2 WindowEd(S) Vectorization

The vectorization of WindowEd(S) is a particular case, as it involves the computation of only 2×2 blocks. Here, vectorization is applied along the antidiagonals using a 128-bit vector (SSE), which is sufficient to handle these antidiagonals. To optimize data storage, the memory layout was reorganized to ensure that each antidiagonal is stored contiguously in memory, effectively eliminating the need for costly gather and scatter operations. Figure S5 illustrates a block diagram

of the vectorized computation. As shown, scalar operations are used for a few words at the beginning and end of the tile until the full width of the SIMD register can be utilized.

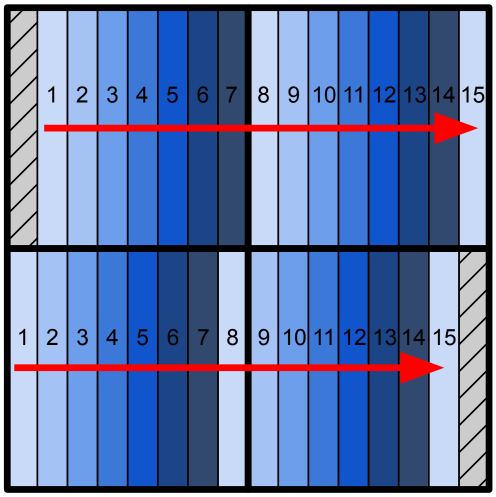

Figure S5: The vectorized implementation of the window computation in the WindowEd(S) component with SSE 128-bit vectors. Words with the same color/number are computed inside the same SSE vector

. Scalar operations are employed for computing the first and last BPM words (dashed) of a window. In the example, each block only contains 8 words.

S7 Empirical Scalability Analysis of QuickEd

In this section, we present an evaluation of QuickEd's empirical scalability compared to classical $O(n^2)$ alignment algorithms' scalability. In practice, QuickEd's pseudopolynomial $O(n\hat{s})$ execution time can achieve better performance than classical polynomial $O(n^2)$ algorithms. We highlight that QuickEd's asymptotic complexity depends on n, the sequence length, and $\hat{s}$. Unlike the strong $O(n^2)$ complexity, which only depends on the sequence length (i.e., irrespectively of $\hat{s}$)), QuickEd's complexity is sensitive to the nominal error between the sequences (or the similarity between the sequences).

Experimental results shown in Figure S6 seek to support this idea quantitatively. We observe that KSW2's $O(n^2)$ time remains unaffected by the alignment score $\hat{s}$ (right panel), whereas QuickEd's $O(n\hat{s})$ time is sensitive to $\hat{s}$ (left panel). As a result, lower $\hat{s}$ (i.e., alignment score or nominal error) yields faster execution for QuickEd. By no means does this result establish sub-quadratic worst-case scaling for QuickEd. Instead, it supports the claim that QuickEd execution time scales better as sequence similarity increases. These results suggest that QuickEd will be particularly useful as sequencing machines advance, producing more accurate sequences with lower error rates.

Similarly, Figure S7 shows QuickEd's execution time across different nominal errors, s. As expected, for a fixed nominal error, QuickEd's execution time scales linearly. This result empirically supports QuickEd's sensitivity to the sequence error and highlights its benefits compared to fixed $O(n^2)$ algorithms (right panel). However, if the alignment score s is proportional to sequence length ($s = e \cdot n$), as is often the case, it follows that $O(\hat{s}n) = O(en \cdot n) = O(n^2)$.

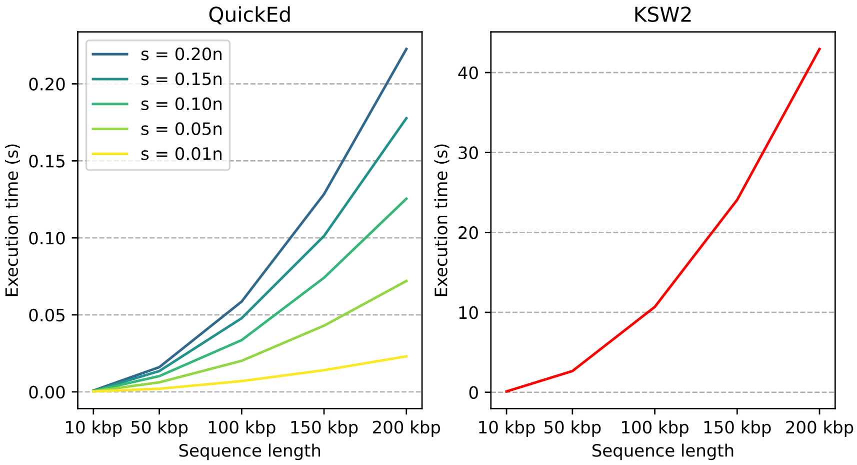

Figure S6: Scalability of QuickEd and KSW2 when aligning sequences ranging from 10 kbp to 200 kbp with an error rate proportional to the sequence length.

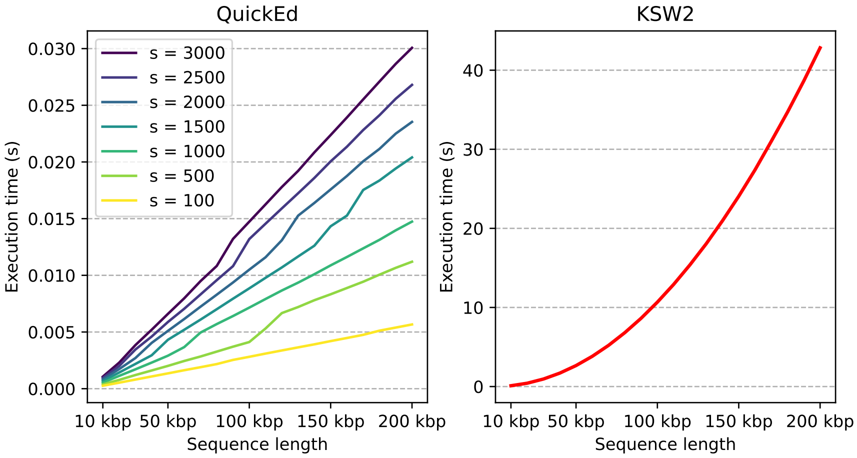

Figure S7: Scalability of QuickEd and KSW2 when aligning sequences ranging from 10 kbp to 200 kbp with a fixed nominal error.

References

Alser, M., Shahroodi, T., Gómez-Luna, J., Alkan, C., and Mutlu, O. (2020). Sneakysnake: a fast and accurate universal genome pre-alignment filter for cpus, gpus and fpgas. *Bioinformatics*, **36**(22-23), 5282–5290.

Bowden, R., Davies, R. W., Heger, A., Pagnamenta, A. T., de Cesare, M., Oikkonen, L. E., Parkes, D., Freeman, C., Dhalla, F., Patel, S. Y., *et al.* (2019). Sequencing of human genomes with nanopore technology. *Nature communications*, **10**(1), 1869.

Daily, J. (2016). Parasail: Simd c library for global, semi-global, and local pairwise sequence alignments. *BMC bioinformatics*, **17**(1), 1–11.

Döring, A., Weese, D., Rausch, T., and Reinert, K. (2008). Seqan an efficient, generic c++ library for sequence analysis. *BMC bioinformatics*, **9**(1), 1–9.

Miga, K. H. and Wang, T. (2021). The need for a human pangenome reference sequence. *Annual Review of Genomics and Human Genetics*, **22**, 81–102.
